# Editorial: In-depth interpretation of critical genomic information related to the biosynthesis of key specialized (secondary) metabolism in medicinal plants

**DOI:** 10.3389/fpls.2025.1680064

**Published:** 2025-09-01

**Authors:** Mingcheng Wang, Xinyi Guo, Zefu Wang

**Affiliations:** ^1^ Institute for Advanced Study, Chengdu University, Chengdu, China; ^2^ Central European Institute of Technology, Masaryk University, Brno, Czechia; ^3^ State Key Laboratory of Tree Genetics and Breeding, Co-Innovation Center for Sustainable Forestry in Southern China, College of Ecology and Environment, Nanjing Forestry University, Nanjing, China

**Keywords:** medicinal plant genomes, specialized metabolic pathway elucidation, multi-omics integration, pre-engineering pathway insights, regulation networks of specialized metabolism

## Introduction

Just over two decades ago, the first plant genome was decoded. Since then, research on specialized metabolites has evolved from labor-intensive phytochemistry into a data-rich, multi-omics enterprise. The 13 articles in this Research Topic illustrate both the impressive advances and ongoing challenges of applying genomics, transcriptomics, metabolomics, and synthetic biology to medicinal plants. Collectively, they investigate 11 species spanning several major metabolite classes, employing strategies ranging from telomere-to-telomere (T2T) genome assemblies to optimized tissue-culture platforms. Above all, these studies demonstrate that modern natural-product science is inherently integrative: pathway genes cannot be fully understood without their regulatory networks, and metabolite accumulation must be interpreted within ecological and developmental contexts.

## Highlights from the Research Topic

The first cluster of contributions demonstrates how chromosome-level genomes (including T2T) unlock complete mechanistic views of specialized metabolic pathways. The T2T assembly of *Hedyotis diffusa* revealed a recent whole-genome duplication that expanded key enzyme families. Through transcriptome analysis, one loganic acid O-methyltransferase and two cytochrome P450 genes were identified as late-stage iridoid tailoring enzymes, closing a critical gap in the biosynthetic map (Chen et al.). Mapping jasmonate-elicited RNA-seq and small RNAs to the *Taxus chinensis* chromosome-level genome identified 990 transcription factors, 460 miRNAs and 160 phasiRNAs linked to paclitaxel biosynthesis. Enzyme genes were most highly expressed in cones and roots, methyl jasmonate failed to induce *GGPPS* or *CoA-ligase*, and the resulting miRNA-phasiRNA network buffered paclitaxel output while suggesting precise engineering targets (Sun et al.). In *Quercus variabilis*, a chromosome-level genome enabled the identification of 22 TCP transcription factors and revealed that *TCP3* is tightly co-expressed with the hydrolysable-tannin glycosyltransferase *UGT84A13* in the cupule. Dual-luciferase assays confirmed that *TCP3* binds and activates the *UGT84A13* promoter, demonstrating how lineage-specific expansion of TCPs governs tissue-specific tannin accumulation (Wang et al.). Combined metabolomic and transcriptomic profiling of *Pogostemon cablin* tissues showed that pogostone primarily accumulates in the roots. Mapping RNA-seq reads to the reference genome, together with expression-metabolite correlation and HXXXD motif screening, highlighted BAHD-DCR acyltransferases as candidate terminal enzymes for pathway engineering (Wang et al.). Twenty-one 2,3-oxidosqualene cyclase (OSC) genes were identified in the *Panax japonicus* genome, and several of these were found to be root-enriched and localized to the nucleus, suggesting specialized roles in tissue-specific triterpenoid biosynthesis (Yang et al.). Finally, analysis of the *Musella lasiocarpa* genome identified 158 WRKY transcription factors distributed across nine chromosomes. Integrating organ-specific RNA-seq and qRT-PCR data, *MlWRKY15*, *MlWRKY111*, and *MlWRKY122* were found to be co-expressed with two O-methyltransferase genes, implicating these WRKYs in organ-specific regulation of phenylphenalenone biosynthesis (Huang et al.). Together, these studies combine high-quality genomes and integrative multi-omics to dissect medicinal plant specialized metabolic pathways with unprecedented resolution, thereby closing critical mechanistic gaps.

The second group of studies showcases what can be achieved when reference genomes are absent. Through integrated transcriptomic and metabolomic profiling, Wu et al. identified seven key enzyme genes and thirteen co-expressed transcription factors that are central to the biosynthesis of Huperzine A in *Huperzia serrata*. The authors further demonstrated that the phenylpropanoid and flavonoid pathways were up-regulated in cultured thalli, a shift that correlated with heightened antioxidant activity and linked metabolite accumulation to improved radical-scavenging capacity. Wang et al. performed targeted metabolomics on the flowers, leaves, and stems of *Aconitum pendulum*, cataloging 198 alkaloids and revealing a broad array of C19/C20 diterpenoid constituents. By integrating these metabolite profiles with tissue-resolved transcriptomes, they constructed a correlation network linking several cytochrome P450s and BAHD acyltransferases to the organ-specific accumulation of aconitine, turupellin, and related diterpenoid alkaloids. Peng et al. performed untargeted LC-MS metabolomics on the leaves, tuberous roots, and fibrous roots of *Tetrastigma hemsleyanum*, revealing pronounced tissue-specific chemical profiles. By integrating these data with RNA-seq, the researchers found that the expression of phenylpropanoid- and isoflavonoid-biosynthetic genes, glucosinolate-pathway enzymes, and several ATP-binding cassette transporters closely tracks the corresponding metabolite distributions. Liu et al. fine-tuned the concentrations of 2,4-dichlorophenoxyacetic acid and 6-benzylaminopurine in *Peucedanum praeruptorum* cultures. The optimized medium increased callus induction to over 85% and rooting success to 69%, while tripling the levels of praeruptorin A, B, and E. Expression of *PpC2*′*H* closely mirrored coumarin accumulation, identifying this hydroxylase as a metabolic bottleneck and a promising target for pathway enhancement. Yerbay et al. examined six high-altitude populations of *Rhodiola linearifolia*, identifying groups that differ sharply in allelic richness and display metabolomic profiles enriched in fatty acids and terpenoids. A strong positive correlation between genetic diversity indices and the abundance of these metabolites highlighted how intraspecific variation drives adaptive metabolic shifts under alpine stress. Together, these studies demonstrate that, even without reference genomes, integrative transcriptomic, metabolomic, and genetic approaches can illuminate specialized metabolic pathways, pinpoint key regulatory and biosynthetic nodes, and guide targeted improvement strategies.

Many of these data-rich studies go beyond component lists to outline actionable engineering strategies. In *Taxus*, the small-RNA network suggests deploying miRNA sponges or CRISPR knock-outs to derepress taxoid P450s (Sun et al.). Overexpressing *TCP3* in *Quercus* could enhance cupule tannin levels (Wang et al.), while tuning specific WRKY factors in *Musella* could increase phenylphenalenone production (Huang et al.). In *Pogostemon*, several BAHD acyltransferases emerged as prime targets for enhancing pogostone (Wang et al.), and in *Peucedanum*, upregulating the C2′H hydroxylase was found to elevate coumarin content (Liu et al.). Thus, these actionable targets provide a roadmap for the future integration of efficient tissue culture and cell factory technologies, laying the groundwork for scalable, high-yield production pipelines.

The two review articles placed these empirical advances within broader methodological and translational contexts. One integrated genomics, transcriptomics, proteomics and metabolomics to reveal how multi-omics dissects biosynthetic gene clusters, pathway reconstruction and stress-response mechanisms in medicinal plants. It also profiled bioinformatics platforms, highlighted single-cell and spatial transcriptomics alongside CRISPR/Cas editing, and identified challenges in data integration, standardization and dynamic pathway mapping for scalable metabolite production (Wang et al.). The second review synthesized current knowledge on benzylisoquinoline alkaloid (BIA) biosynthesis by cataloging key enzymatic steps from norcoclaurine synthase to P450 monooxygenases and integrating multi-omics findings to close pathway gaps. It evaluated synthetic biology strategies such as modular reconstruction in microbial and plant hosts along with dynamic flux control for scalable BIA production (Zhao et al.).

## Summary and future perspectives

This Research Topic highlighted two complementary paradigms for dissecting medicinal plant metabolism. Genome-enabled studies exploit chromosome-level and T2T assemblies to uncover duplications, gene family expansions and regulatory networks, identifying the enzymes and transcription factors that drive tissue- and stress-specific metabolite accumulation. Parallel efforts using *de novo* transcriptomes, metabolite profiling and genetic markers chart biosynthetic modules and post-transcriptional controls even in species lacking reference assemblies. Together, these approaches deliver a coherent set of strategies for identifying bottlenecks and engineering targets across various metabolite classes.

Looking ahead, realizing the full complexity of plant specialized metabolism will require integrating single-cell methods to resolve cell-type pathways, spatial omics to trace metabolite flux *in situ*, and artificial intelligence-powered models to predict enzyme function and network dynamics. Coupling these innovations with high-quality genomes and biochemical validation will transform pathway elucidation from a retrospective description to a forward-looking design. These advances will enable the scalable, sustainable production of high-value natural products and guide the discovery of novel bioactive compounds.

